# Structure–Property–Function Evaluation of a β-Type Ti-Nb-Zr Alloy for Dental Implant Applications with Short-Term Clinical Validation

**DOI:** 10.3390/jfb17020096

**Published:** 2026-02-14

**Authors:** Deukwon Jo, Soo-Hwan Byun, Sang-Yoon Park, Jong-Hee Kim, Mijoo Kim, Hyo-Jung Lee, Young-Kyun Kim, Byoung-Eun Yang, Yang-Jin Yi

**Affiliations:** 1 Section of Restorative Dentistry, Division of Preventive and Restorative Sciences, Los Angeles School of Dentistry, University of California, 714 Tiverton, Los Angeles, CA 90095, USA; deukwonjo@dentistry.ucla.edu (D.J.); mijookim@dentistry.ucla.edu (M.K.); 2 Department of Oral and Maxillofacial Surgery, Hallym University Sacred Heart Hospital, Anyang 14066, Republic of Korea; purheit@daum.net (S.-H.B.); psypjy0112@naver.com (S.-Y.P.); 3 Dental AI-Robotics Center, Hallym University Sacred Heart Hospital, Anyang 14066, Republic of Korea; 4 Department of Artificial Intelligence and Robotics in Dentistry, Graduate School of Clinical Dentistry, Hallym University, Chuncheon 24252, Republic of Korea; 5 Department of Prosthodontics, Section of Dentistry, Seoul National University Bundang Hospital, Seongnam 13620, Republic of Korea; pcbs98@snubh.org; 6 Department of Periodontology, Section of Dentistry, Seoul National University Bundang Hospital, Seongnam 13620, Republic of Korea; periolee@gmail.com; 7 K Oral and Maxillofacial Surgery Dental Private Office, Seongnam 13637, Republic of Korea; kyk0505@daum.net; 8 Department of Dentistry & Dental Research Institute, School of Dentistry, Seoul National University, Seoul 03080, Republic of Korea

**Keywords:** Ti-Nb-Zr alloy, dental implants, β-type titanium, fatigue resistance, elastic modulus, fractal dimension, peri-implant bone adaptation, functional biomaterials

## Abstract

Titanium-based alloys are widely used in dental implantology; however, the mechanical limitations of commercially pure titanium (cpTi) and unresolved concerns regarding stress shielding remain. This study evaluated the structure–property–function relationship of a novel β-type titanium-niobium-zirconium (Ti-Nb-Zr; TNZ) alloy for dental implant applications. Laboratory testing assessed the elemental composition, tensile properties, and fatigue resistance of the cpTi, compared with modified Grade 4 cpTi (MG4T). In parallel, a randomized, single-blind, controlled clinical trial was conducted over 12 months to compare the clinical performance of TNZ and MG4T implants under functional loading. A total of 80 participants (mean age: 54.2 years; 43 females, 37 males) were enrolled, with 77 completing the 12-month follow-up (TNZ: *n* = 38; MG4T: *n* = 39). Clinical outcomes included implant success and survival, peri-implant soft tissue parameters, marginal bone levels, fractal dimension (FD) analysis of trabecular bone, and adverse events. TNZ implants demonstrated superior fatigue resistance without an increase in the elastic modulus relative to MG4T. Clinically, both groups achieved 100% implant success and survival, with no implant-related adverse events. FD analysis revealed time-dependent bone remodeling without evidence of pathological adaptation. These findings support the functional viability of TNZ as a mechanically robust, biocompatible implant material. Further long-term, multicenter trials are warranted to confirm sustained clinical benefits and broader applicability.

## 1. Introduction

Dental implants have become a primary solution for tooth replacement, prompting extensive research into optimizing the biological and technical properties of implant materials. The most commonly utilized materials are commercially pure titanium (Grade 4, cpTi) and the titanium alloy Titanium-6Aluminum-4Vanadium (Grade 5, Ti-6Al-4V), due to their biocompatibility, mechanical strength, and proven clinical outcomes [1,2,3]. However, these materials present certain limitations. In particular, Ti-6Al-4V is associated with the release of aluminum and vanadium ions, which have been linked to cytotoxic and pro-inflammatory effects [4,5,6]. To address these concerns and account for the oral cavity’s aggressive environment, many manufacturers have transitioned to G4 cpTi to improve long-term stability and reduce ion release. To offset the lower mechanical strength of G4 cpTi relative to Ti-6Al-4V, a cold-worked variant known as modified Grade 4 titanium (MG4T) was introduced and has since been widely adopted in dental implant manufacturing [6].

Despite these advancements, implant fractures, such as bending and cracking, have been documented [7,8]. Although implant fractures are uncommon in clinical practice [9], they constitute significant functional challenges when they occur. Specific clinical scenarios, including the placement of narrow implants in patients with parafunctional habits or within regions with limited bone volume, particularly in the posterior jaw, further elevate the risk of failure [9,10,11]. These demanding conditions highlight the need to enhance the mechanical robustness of implant materials to withstand cyclic loading.

For these reasons, various Ti alloys have also been investigated and tested for clinical use [1,2,5,6,7,12,13,14,15,16]. In both the orthopedic and dental fields, the focus of biomaterials research has shifted toward developing alloys that eliminate cytotoxic elements, such as aluminum and vanadium, while concurrently enhancing mechanical strength and fatigue resistance to meet long-term load-bearing demands [15,17].

In developing new alloys, researchers and developers have considered the stress-shielding effect of materials on the surrounding bone. The relationship between stress distribution and implant materials is closely tied to their elastic modulus. Materials with a higher elastic modulus tend to absorb more stress, whereas those with a lower modulus transfer more of that stress to the surrounding bone. As a result, when an implant’s modulus of elasticity is too high, it can prevent proper load transfer, leading to peri-implant bone atrophy [18,19]. This phenomenon is known as the stress-shielding effect in orthopedics [10,15,20,21]. In this context, elastic modulus is considered a major factor influencing the mechanical environment at the bone–implant interface and subsequent bone adaptation processes [22].

Several β-type titanium alloys, such as the Titanium-Niobium-Zirconium (Ti-Nb-Zr; TNZ) system, have recently been developed to address this issue by lowering the elastic modulus while maintaining strength [23]. Contemporary alloy development emphasizes β-type titanium systems that integrate high strength with a low elastic modulus. This is accomplished by alloying titanium with biocompatible elements such as niobium, zirconium, and tantalum. For instance, Calazans Neto et al. reviewed the emergence of TNZ alloys, which are engineered to possess an elastic modulus approximating that of cortical bone. This strategy preserves tensile strength and enhances cytocompatibility, positioning these alloys as potential candidates for orthopedic and dental applications [3]. By minimizing the elastic modulus mismatch between dental implants and bone, TNZ alloys provide a solid platform for studying mechanically induced bone responses in dental implantology [24].

Orthopedic research on bone remodeling and implant stiffness has indicated a relationship in which stiffer implant materials tend to slow down and irregularize the bone-healing process [15]. These findings have motivated the development of implant materials that emphasize mechanical compatibility, characterized by a lower elastic modulus combined with sufficient strength to support physiologic load transfer.

In line with these efforts, TNZ alloys were developed for orthopedic devices. These alloys are particularly notable for their high elastic admissible strain, excellent mechanical properties, and outstanding cytocompatibility [16]. Ozan et al. evaluated different compositions of Ti, Nb, and Zr, measuring their tensile strength, elastic modulus, elongation at rupture, and elastic admissible strain, which ranged from 704 to 839 MPa, 62 to 65 GPa, 9.9% to 14.8%, and 1.08% to 1.31%, respectively [16]. Notably, its elastic modulus is significantly closer to that of cortical bone (27 GPa) [25] compared to the Ti-6Al-4V alloy (110 GPa) [14], underscoring its capacity for enhanced mechanical compatibility. Accordingly, there has been growing interest in the application of TNZ alloys in dental implantology [3].

On the other hand, questions have also arisen about whether adopting the stress-shielding concept from research on reinforced long bones is appropriate for implant dentistry [20,21]. Because of their unique loading patterns and biomechanical context, stress shielding may play a less significant role in implant dentistry compared to orthopedics. According to experts such as Wiskott and Belser, the most important factors for successful osseointegration are surface texture and biomechanical coupling. Their work suggests that improving an implant’s surface characteristics should be the primary priority, rather than simply matching its stiffness to that of the bone [21]. Meanwhile, Korabi et al. suggested, based on their numerical simulations, that stiffer implants performed better biomechanically and allowed the safer application of higher loads, thereby challenging traditional stress-shielding concerns [20].

In implant dentistry, no consensus has been reached to date regarding the impact of implant materials with an elastic modulus closer to that of bone on implant-surrounding bone remodeling during function. Recently, a dental implant using TNZ alloy has been developed; however, no clinical trials involving these implants have been reported until now. No clinical investigations have yet confirmed the safety and performance of TNZ alloys in dental implantology, despite their encouraging cytocompatibility and mechanical properties in orthopedic research [16]. Establishing such translational evidence is therefore essential for evaluating TNZ alloys as candidate functional biomaterials for dental implants.

Accordingly, this study was designed to (1) verify the composition and mechanical properties of the newly developed TNZ alloy through mechanical testing; (2) assess the functional and biological feasibility of TNZ dental implants in comparison with MG4T implants within a randomized controlled clinical trial; and (3) analyze and compare changes in fractal dimension (FD) of peri-implant bone at multiple time points to investigate potential material-dependent differences in bone microarchitectural adaptation during functional loading.

## 2. Materials and Methods

The test group consisted of implants fabricated from a titanium-niobium-zirconium alloy (WPM-TSIII SA, Osstem Implant Co., Seoul, Republic of Korea), hereafter referred to as TNZ implants. The control group used implants made of modified Grade 4 commercially pure titanium (TSIII SA, Osstem Implant Co., Seoul, Republic of Korea), hereafter referred to as MG4T implants.

### 2.1. Surface and Mechanical Experiment

Surface and mechanical experiments were conducted on a representative specimen to verify the composition and mechanical properties of the TNZ implant materials. These experiments were designed to assess functional properties comparatively under clinically relevant conditions, rather than to determine absolute mechanical limits. Raw data were directly reported in the Results section without further processing.

#### 2.1.1. Surface Composition Analysis

To confirm the elemental composition of the newly developed TNZ implants, energy-dispersive spectroscopy (EDS) was performed. Three implant fixtures (4.0 mm in diameter and 11.0 mm in length) composed of the TNZ alloy were analyzed using an energy-dispersive spectrometer (INCA Energy, Oxford, UK) integrated with a scanning electron microscope (JSM-7016F Plus, JEOL, Tokyo, Japan). The analysis was conducted at a magnification of 500×. EDS was employed to confirm the elemental composition of the alloy. Phase constitution analysis was beyond the scope of this assessment.

#### 2.1.2. Tensile Strength, Elastic Modulus, and Elongation

**Figure 1:**
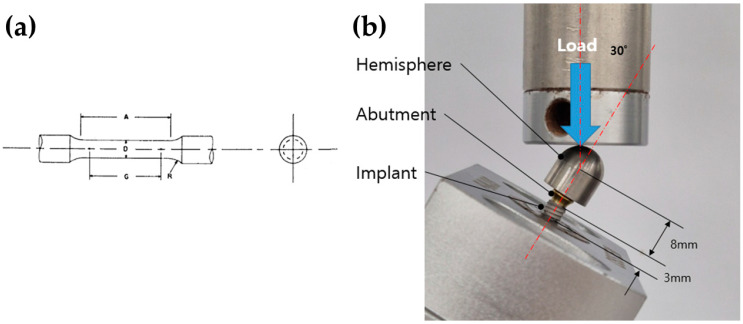
(**a**) Schematic diagram of the standardized tensile specimen conforming to ASTM E8/E8M-22 geometry. (**b**) Photograph of machined implant-abutment-hemisphere assemblies prepared for dynamic mechanical testing. The blue arrow indicates the direction of applied compressive load, and the red dashed line denotes the 30° angulation between the implant axis and the loading direction, consistent with ISO 14801. The distances from the implant platform to the loading center and from the fixture base to the platform are 8 mm and 3 mm, respectively.

Five specimens from each group (MG4T and TNZ: Osstem Implant Co., Seoul, Republic of Korea) were prepared in accordance with ASTM E8/E8M-22: Standard Test Methods for Tension Testing of Metallic Materials [26]. The specimens had a gauge length of 11.0 ± 0.1 mm, a diameter of 2.5 ± 0.1 mm, a fillet radius of 2 mm, and a reduced section length of 16 mm, conforming to the standard specimen geometry specified in ASTM E8/E8M-22 (Figure 1a). Tensile testing was performed using a universal testing machine (Instron 8871, Instron, Norwood, MA, USA). The tensile tests were intended to compare the elastic and plastic deformation behavior of the materials under standardized loading conditions. Young’s modulus was calculated by linear regression of the initial elastic portion of the stress–strain curve. Specifically, a strain range of 0.002–0.005 (0.2–0.5%) was applied in accordance with ASTM E111-17 [27] and optimized for the present experimental setup. This range was selected to reflect the most linear and noise-free region of the stress–strain curve while avoiding the onset of plastic deformation. This approach is consistent with methods used in prior studies evaluating titanium-based biomaterials under comparable testing conditions [28].

#### 2.1.3. Fatigue Test

Fatigue testing was conducted in accordance with ISO 14801: Dynamic Loading Test for Endosseous Dental Implants [29]. Five identical fixtures (4.0 mm × 11.0 mm) were prepared for both the MG4T and TNZ groups. As depicted in Figure 1b, each fixture was mounted onto a metal collet, and a corresponding non-hex abutment with a diameter of 5.0 mm was secured using a torque of 30 Ncm. A hemispherical cap was positioned on the abutment to apply the load. Testing was performed using a fatigue failure testing machine (Instron E3000, Instron, Norwood, MA, USA), set to a minimum load of 40 N and a maximum load of 400 N at a frequency of 14 Hz. At 1000 cycles, displacement differentials between the 40 N and 400 N load levels were recorded to evaluate elastic compliance under cyclic stress. This protocol was employed as a screening-level comparative fatigue assessment under identical testing conditions. Fatigue failure was defined as a displacement exceeding 1.5 times the predetermined baseline displacement difference. This criterion was established to detect the onset of functional failure—characterized by significant loss of system stiffness due to crack propagation, screw loosening, or severe plastic deformation—prior to catastrophic fracture or equipment damage [29,30]. Although five specimens per group were tested, no formal statistical analysis was performed due to the exploratory design of this fatigue assessment. Given the limited sample size, results were interpreted as qualitative indicators of fatigue behavior under standardized conditions, rather than definitive measures of mechanical superiority.

### 2.2. Randomized Controlled Clinical Trial

#### 2.2.1. Study Design

This prospective, multicenter, randomized (1:1), parallel-arm, single-blind (participant-blinded), pivotal clinical trial was conducted at two sites: Seoul National University Bundang Hospital (Seongnam, Republic of Korea) and Hallym University Sacred Heart Hospital (Anyang, Republic of Korea). The study was approved by the Ministry of Food and Drug Safety (MFDS) of the Republic of Korea (Approval No. 951) and conducted in accordance with the Declaration of Helsinki. Institutional Review Board (IRB) approval was obtained at both centers (IRB No. E-1909/564-003 and 2020-12-011, respectively), including approval of the protocol, informed consent form, and all study-related materials.

The clinical trial used a single-blind design, as it was not feasible to blind the operators because of visible differences between implant types. To maintain objectivity, an independent contract research organization (CRO) oversaw all study procedures, collected and anonymized patient data, and provided the information to blinded outcome assessors and statisticians. Outcome data and adverse event records were collected and analyzed independently by the CRO. The study was conducted in compliance with CONSORT guidelines. Furthermore, the trial was registered at the Korea Disease Control and Prevention Agency (https://cris.nih.go.kr/cris/search/detailSearch.do?seq=23920&search_page=L: Registration No., KCT0006874, accessed on 12 October 2025).

Because the TNZ implant was not yet commercially available at the time of the study, the clinical trial was designed to evaluate functional comparability and biological safety relative to an established implant system, rather than to establish clinical superiority. The test implant (TNZ) and the control implant (MG4T) shared identical fixture designs, dimensions, and prosthetic components. Both implant systems featured an internal connection with platform switching, a tapered body, a self-tapping cutting edge, and sandblasted, large-grit, acid-etched surfaces. This design equivalence allowed the fixture material composition to be examined as the primary experimental variable. The sole distinction between the two groups was the fixture material composition: the TNZ group used a titanium alloy containing niobium (39–41%) and zirconium (5–8%), whereas the control group used modified Grade 4 commercially pure titanium (cpTi). All implants used in the clinical trial were terminally sterilized using gamma irradiation in accordance with ISO 11137 guidelines for medical devices [31]. Due to visible differences in implant appearance, blinding of operators was not feasible; thus, the study adopted a single-blind design, blinding only the participants. Allocation concealment was ensured using opaque, sealed envelopes. Independent, blinded evaluators conducted all outcome assessments, and an external CRO supervised all procedures per GCP.

#### 2.2.2. Participants’ Eligibility and Clinical Workflow

Screening and selection of participants were conducted according to predetermined inclusion and exclusion criteria. Eligible individuals were adults aged 19 years or older with fully developed jawbones and one to three consecutive missing premolars or molars (FDI tooth numbering system; 14–18, 24–28, 34–38, 44–48) requiring implants. Additional eligibility criteria included the presence of opposing dentition (either natural or implant-supported), no planned implant placements in adjacent or opposing teeth, sufficient anatomical suitability, bone quality verified by CBCT, and a residual bone height of at least 5 mm in cases requiring sinus lift. Written informed consent and agreement to comply with trial protocols were mandatory.

Exclusion criteria included heavy smoking (≥20 cigarettes per day), severe parafunctional habits, poor oral hygiene, impaired wound healing, or contraindications to oral surgery. Individuals taking medications that affect bone metabolism, such as bisphosphonates or corticosteroids for more than seven days within the previous six months, were excluded. Additional exclusions applied to immunocompromised individuals, those with recent bone grafts or mucosal lesions, and those with systemic conditions such as uncontrolled diabetes or recent myocardial infarction. Individuals with allergies to implant materials, pregnant participants, those enrolled in other clinical trials, or those deemed unsuitable by the investigator were also excluded.

**Figure 2:**
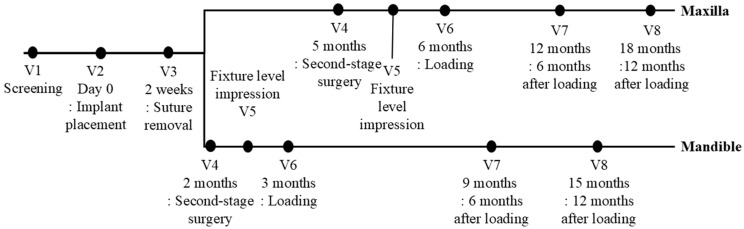
Timeframe for the randomized controlled clinical trial. V = Visit.

Baseline assessments for eligible participants included panoramic and periapical radiographs as well as CBCT imaging (Visit 1). Implant placement (Visit 2) was performed according to institutional protocols and manufacturer guidelines, with an insertion torque target of 30–50 Ncm. ISQ values were measured using the Osstell Mentor device. Participants returned for suture removal (Visit 3), followed by second-stage surgery at two months for mandibular implants or five months for maxillary implants (Visit 4). Cases with an ISQ below 60 were withdrawn from the study. Fixture-level impressions were taken at Visit 5, and final prostheses were delivered at Visit 6. Follow-up assessments were conducted at 6 and 12 months post-loading (Visits 7 and 8). Screening, surgical, prosthetic, and follow-up visits are illustrated in Figure 2.

#### 2.2.3. Primary Outcome Measurements

The primary outcome of the clinical trial was the implant success rate at 12 months following functional loading. This endpoint was selected to confirm the absence of adverse functional or biological responses during early service. Implant success was defined according to established criteria [32,33] and included: (1) absence of persistent pain, foreign body sensation, or abnormal sensory perception; (2) absence of signs of inflammation with suppuration on periodontal probing; (3) probing depth less than 5 mm with no bleeding on probing; (4) absence of implant mobility; and (5) radiographic evidence of no radiolucency surrounding the implant, with marginal bone loss limited to ≤1 mm during the follow-up period.

#### 2.2.4. Secondary Outcome Measurements

Secondary outcomes comprised the implant success rate at 6 months post-loading and the implant survival rates at 6 and 12 months post-loading. Implant survival was defined as the implant remaining in situ without loss, irrespective of marginal bone loss or complications. Survival rates were determined as the proportion of surviving implants relative to the total number of implants placed.

At the 6-month and 12-month follow-up visits, additional clinical parameters were recorded, including probing depth (PD), plaque index (PI), gingival index (GI; Löe and Silness), and bleeding on probing (BOP). Marginal bone level (MBL) changes and FD values around the implants were also evaluated using standardized periapical radiographs (PAs). All radiographs were assessed on a 27-inch IPS FHD monitor by two calibrated observers with 20 and 16 years of clinical experience, respectively.

MBL assessments were conducted on the mesial and distal surfaces of each implant by measuring the vertical distance from the implant platform to the most coronal point of bone-to-implant contact. Measurements were obtained at crown delivery (Visit 6), 6 months post-loading (Visit 7), and 12 months post-loading (Visit 8). Bone level changes were categorized as ‘maintained’ or ‘decreased,’ with recognition that image distortion from elongation or foreshortening on PAs may lead to misestimation of actual bone level changes [34,35,36,37,38]. Increases in bone level were not categorized as a clinical endpoint. If a decrease in bone level greater than 1 mm was observed at either follow-up, the implant was excluded from the success count but remained in the survival analysis if other success criteria were met. To standardize measurements, marginal bone loss was recorded as 1.1 mm if the bone level fell below the peak of the first implant thread, as specified by the manufacturer.

#### 2.2.5. Sample Size and Non-Inferiority Margin Determination

This clinical trial was designed to demonstrate that the implant success rate in the test group (TNZ) was not inferior to that in the control group (MG4T) among patients undergoing masticatory rehabilitation. The primary endpoint was the 12-month post-loading success rate.

A retrospective study by Kim et al. reported success rates of 96.6% and 91.2% for implants in the maxillary and mandibular molar regions, respectively [39]. Consequently, a conservative estimated success rate of 96.6% was adopted for both test and control implants. Supporting literature includes Tolentino et al., who reported a 95.2% success rate in 42 subjects [33], and Wang et al., who reported a 100% success rate in 30 subjects [40]. In contrast, Deporter et al. observed a lower rate of 83.3% in a 46-subject trial [41]. Based on these values, a non-inferiority margin of 13.3%—the difference between 96.6% and the lowest documented rate of 83.3%—was predefined as clinically acceptable based on prior literature. Using this margin, a one-sided significance level of 0.025, and a power of 85%, the required sample size was calculated as 34 participants per group. To account for a potential 15% dropout rate, the final sample size was set at 40 participants per group, for a total of 80 participants.

#### 2.2.6. Adverse Events (AEs)

Adverse events (AEs) were monitored throughout the trial. Treatment-emergent adverse events (TEAEs) were defined as any unfavorable sign or symptom occurring after implant placement, irrespective of causality. Adverse events were classified according to the Medical Dictionary for Regulatory Activities (MedDRA; MSSO, McLean, VA, USA), based on System Organ Class and Preferred Term. Each AE was evaluated by study investigators for severity (mild, moderate, severe), seriousness (serious or non-serious), and causality (related, possibly related, unrelated). Serious adverse events (SAEs) were defined as events resulting in death, being life-threatening, requiring hospitalization or prolonged hospitalization, causing significant disability or incapacity, or leading to congenital anomalies. In cases of SAEs, immediate reporting to the Institutional Review Board and regulatory authorities was conducted. An independent contract research organization (CRO) supervised the safety monitoring process, ensuring unbiased data collection, coding, and reporting. The CRO also ensured adherence to Good Clinical Practice (GCP) and national regulatory requirements.

### 2.3. Fractal Dimension Analysis

FD analysis was conducted on standardized periapical radiographs collected at four time points: second-stage surgery (Visit 4; T0), prosthesis delivery (Visit 6; T1), and at 6 months (Visit 7; T2) and 12 months (Visit 8; T3) post-loading. This exploratory approach aimed to identify potential material-dependent differences in peri-implant bone microarchitecture during functional loading. All analyses utilized validated software (Quvrad, version 1.6; QUVE Seventeen, Seoul, Republic of Korea) specifically designed for FD evaluation around dental implants.

**Figure 3:**
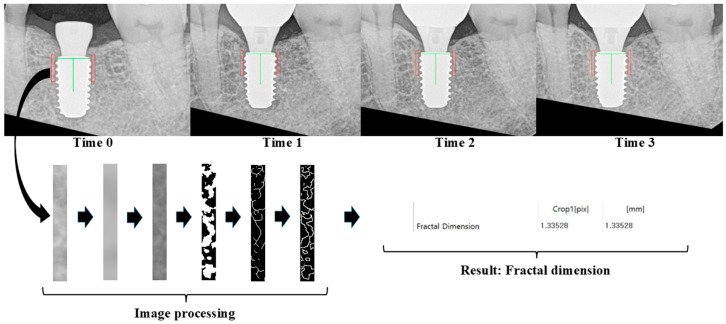
Fractal analysis was conducted using QuvRAD software (version 1.6; Quve Seventeen, Seoul, Republic of Korea). To analyze periapical radiographs from four time points (Time 0: Visit 4, Time 1: Visit 6, Time 2: Visit 7, and Time 3: Visit 8), all images were first recentered and reoriented to ensure that the target implant appeared upright and centered. The green vertical line indicates the long axis of the implant, and the red rectangles represent the regions of interest (ROIs) selected for trabecular analysis. The image processing protocol followed White and Rudolph’s method, which includes blurring, subtraction, gray-level normalization (adding a gray value of 128), binarization, and skeletonization. The arrows indicate the sequential steps of image processing and fractal dimension calculation.

Figure 3 demonstrates that the software standardized the orientation of each periapical image and identified a region of interest (ROI) in the mesial and distal bone adjacent to the implant. The ROI was defined relative to the actual implant fixture diameter to ensure measurement consistency across all time points. Vertically, the ROI extended from just above the implant platform to the fourth fixture thread; horizontally, it included bone adjacent to, but not overlapping, the fixture threads. This standardized ROI definition was implemented to improve longitudinal comparability rather than to measure absolute trabecular complexity.

Image processing followed the protocol established by White and Rudolph [42], which included grayscale conversion, Gaussian filtering, binarization, and skeletonization. FD values were automatically calculated using a box-counting algorithm integrated into the software.

ROI placement was informed by previous finite element analyses of peri-implant stress distribution [22,43,44], to target bone regions anticipated to undergo adaptive remodeling in response to mechanical stimuli.

### 2.4. Statistical Analysis

All statistical analyses, except those related to FDs, were performed using SAS 9.4 (SAS Institute Inc., Cary, NC, USA). For the primary outcome, non-inferiority was established if the difference in implant success rates (test group minus control group) at 12 months post-loading exceeded −13.3%, the predefined non-inferiority margin, as determined by the lower limit of the one-sided 97.5% confidence interval.

For secondary outcomes and adverse events, between-group comparisons were conducted using independent two-sample *t*-tests for normally distributed variables or Wilcoxon rank-sum tests when normality was not met. Within-group changes were analyzed using paired *t*-tests or Wilcoxon signed-rank tests, depending on data distribution. For categorical data, Chi-square tests were applied unless more than 20% of expected frequencies were less than 5, in which case Fisher’s exact test was used.

Interobserver agreement for radiographic measurements, including marginal bone levels and FDs, was assessed using the intraclass correlation coefficient (ICC) with a two-way random effects model. FD data were further analyzed using paired *t*-tests and linear mixed-effects models. These analyses were performed using R version 4.3.0 (The R Foundation for Statistical Computing, www.r-project.org). All other statistical tests were two-sided with a significance threshold of *p* < 0.05.

## 3. Results

### 3.1. Surface and Mechanical Experiments

**Figure 4:**
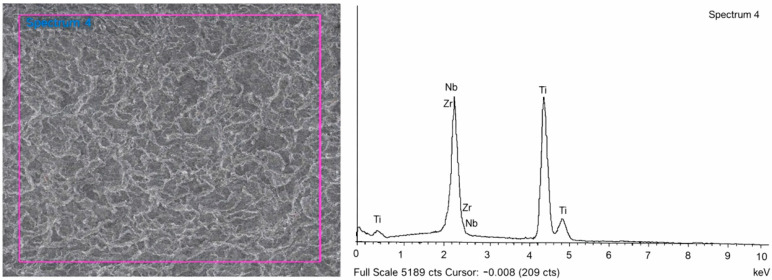
SEM image and energy-dispersive X-ray spectroscopy (EDS) pattern for TNZ implants (WPM-TSIII SA). The purple frame indicates the region of interest selected for EDS analysis.

Table 1 and Figure 4 present the results of surface composition analysis for TNZ implants in the test group, as determined by energy-dispersive X-ray spectroscopy (EDS). The EDS data indicate a consistent and homogeneous distribution of titanium, niobium (40.12–41.11 wt%), and zirconium (7.10–7.49 wt%) across the analyzed surfaces. Scanning electron microscopy (SEM) images reveal distinct morphological differences between the two alloys. MG4T samples display relatively coarse-grained structures characteristic of cold-worked α-phase titanium, while TNZ samples exhibit a finer and more homogeneous microstructure indicative of β-phase morphology. No surface defects or segregations were observed in either group. The uniform dispersion of Nb and Zr in TNZ specimens demonstrates the alloy’s compositional stability and suggests that the homogeneous alloy distribution may contribute to improved fatigue resistance and long-term mechanical integrity.

Table 2 summarizes the mechanical properties, including tensile strength, elastic modulus, and elongation, for both MG4T and TNZ materials. Table 3 provides the raw fatigue-strength data for each implant type. Due to the limited sample size, statistical comparisons were not conducted. Nevertheless, the results indicate that TNZ demonstrates higher tensile strength and fatigue resistance under the tested conditions, without a corresponding increase in elastic modulus compared to MG4T.

### 3.2. Randomized Controlled Clinical Trial Results

**Figure 5:**
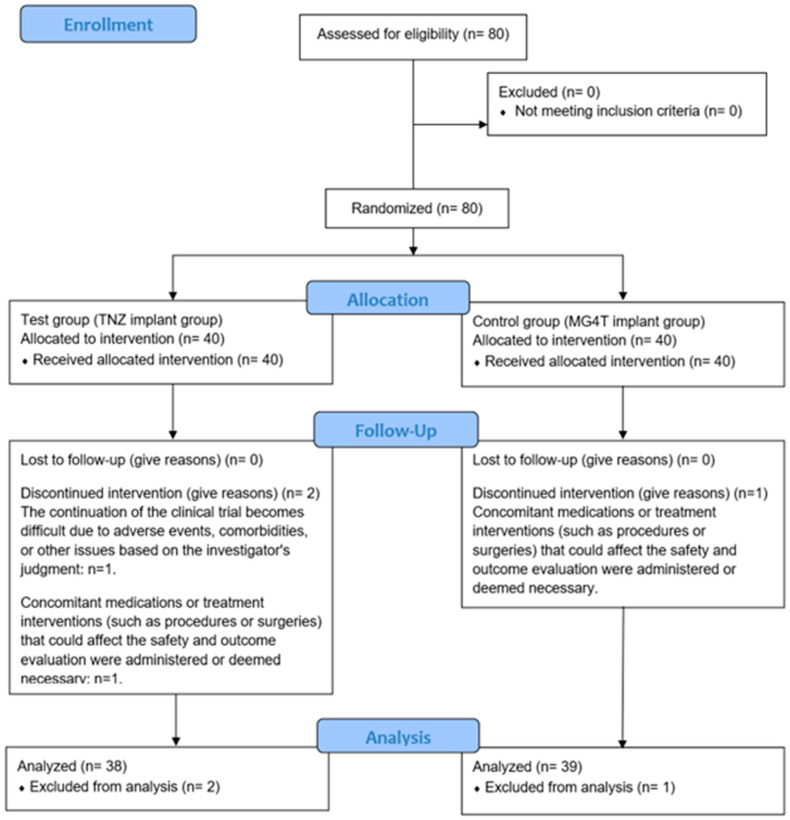
Flow diagram of the test group (TNZ) and the control group (MG4T).

Eighty participants requiring dental implants for esthetic and functional rehabilitation were screened. All participants provided written informed consent and met the inclusion criteria. Eligible individuals were randomly allocated to either the test group (*n* = 40) or the control group (*n* = 40). Of these, 77 participants (96.25%) completed the clinical trial according to protocol (test: 38 [95.00%]; control: 39 [97.50%]). One participant from each group underwent bone grafting at the time of implant placement, which constituted a protocol deviation and led to exclusion. An additional participant in the test group was withdrawn following a diagnosis of a salivary gland tumor. Participant progression and reasons for withdrawal are presented in the CONSORT flowchart (Figure 5). Baseline demographic and clinical characteristics prior to loading are summarized in Table 4.

#### Primary and Secondary Outcomes

At 12 months post-loading, both groups demonstrated 100% implant success. The one-sided 97.5% lower confidence interval for the between-group difference surpassed the predefined non-inferiority margin. Success and survival rates remained at 100% throughout the follow-up period. Peri-implant soft tissue parameters, including probing depth, plaque index, gingival index, and bleeding on probing, were assessed at 6 and 12 months. At 12 months, the gingival index was significantly lower in the test group than in the control group (*p* = 0.0454), no other differences reached statistical significance (Table 5). Marginal bone levels were evaluated on periapical radiographs by two blinded observers. All implants maintained marginal bone levels, with no observed bone loss greater than 1 mm at follow-up.

### 3.3. Fractal Dimension Analysis Results

**Figure 6:**
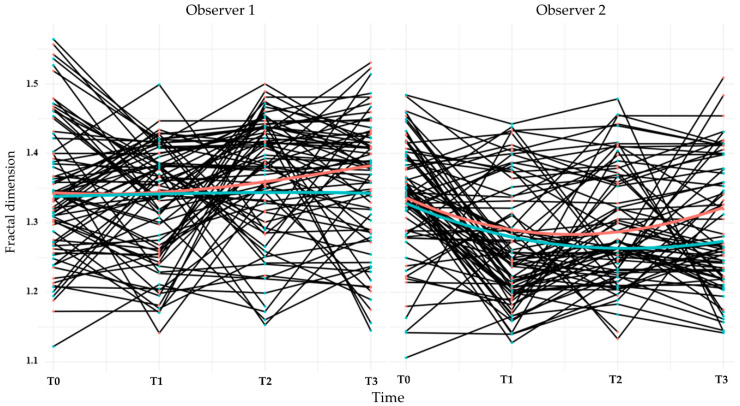
Line plots showing the longitudinal changes in fractal dimension for each participant over the clinical trial. The black lines represent individual patient trajectories. The red line indicates the average for the control group (MG4T), and the green line indicates the average for the test group (TNZ). Data are shown separately for Observers 1 and 2, with measurements recorded at four time points (T0-T3).

**Figure 7:**
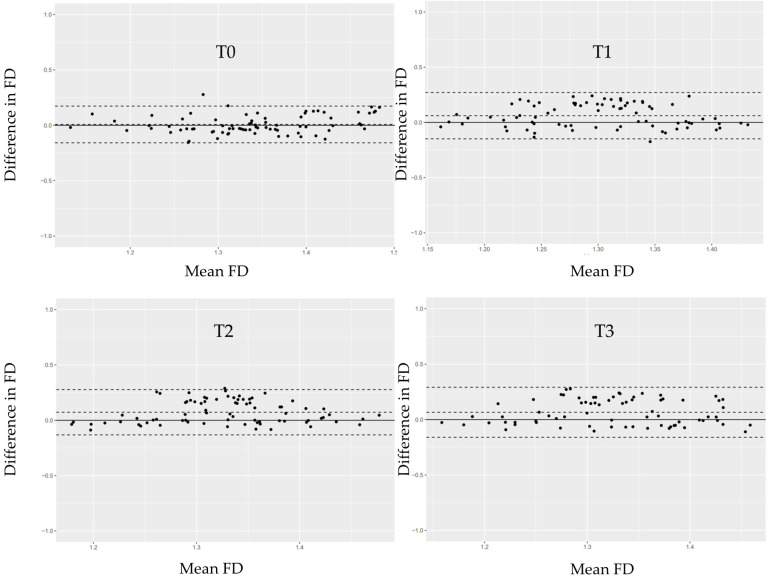
Bland–Altman plots illustrating the differences in fractal dimension (FD) measurements between Observer 1 and Observer 2 at each time point (T0–T3). The solid horizontal line represents the mean difference between the two observers. The dashed lines indicate the limits of agreement (mean ± 1.96 × SD). Each black dot represents an individual participant’s paired measurement.

FDs were evaluated at four time points: second-stage surgery (T0), prosthesis delivery (T1), and at 6 (T2) and 12 (T3) months post-loading. ROIs were selected adjacent to the implant threads, as shown in Figure 6. Mean FD values and interobserver intraclass correlation coefficients (ICCs) are provided in Table 6 and Table 7, respectively. ICCs were acceptable for all time points except T0. Bland–Altman plots (Figure 7) confirmed good agreement between observers.

**Figure 8:**
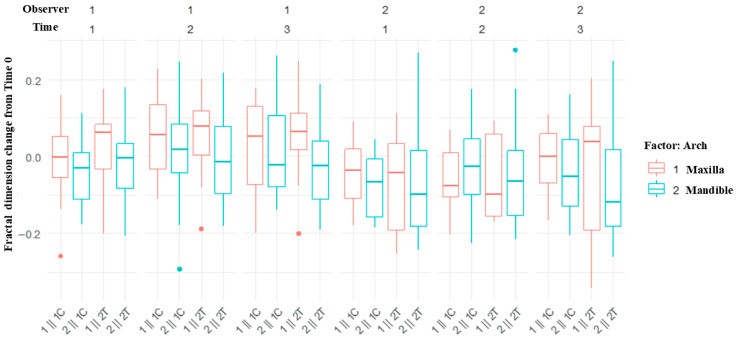
Box plots showing fractal dimension changes from Time 0 in the maxilla and mandible. The label on the x-axis represents the arch, observer, and control or test group. For example, 1ǁ 1C indicates the maxilla, observer 1, and the control group. Control group (MG4T), Test group (TNZ).

FDs decreased or remained stable from T0 to T1 and subsequently increased at T2 and T3. Observer 2’s measurements showed statistically significant reductions at T1 and T2 compared with baseline, except at T3 in the control group (Table 8). Linear mixed model analysis (Table 9) indicated a significant negative association between baseline FD and FD change over time (*p* < 0.001). Between-group differences were not detected at T1 and T2 (*p* > 0.05) but were observed at T3 (*p* = 0.016). Sex was associated with FD at T1 (*p* = 0.022), whereas age was not associated with FD changes. FD changes appeared more pronounced in the maxilla (Figure 8); however, statistical analysis revealed no significant differences between jaws at any time point.

### 3.4. Adverse Events

Adverse events (AEs) were evaluated in the safety population (*n* = 80; 40 per group). In the test group, one target-site AE (toothache) was observed and resolved without intervention. No target-site AEs were reported in the control group. No statistically significant differences in AE incidence were found between groups.

Nine non-target-site AEs were reported in seven participants in the test group (17.5%), while 11 non-target-site AEs occurred in eight participants in the control group (20.0%). One serious adverse event (salivary gland tumor) was reported in the test group and was determined to be unrelated to the implant. No device-related AEs or serious adverse events (SAEs) were identified. The incidence of AEs did not differ significantly between groups. All AEs were classified and reported using MedDRA terminology.

## 4. Discussion

This study consisted of two primary components: a mechanical evaluation of the TNZ alloy’s composition and mechanical properties, and a randomized controlled clinical trial comparing the clinical performance of TNZ implants with MG4T implants over a 12-month loading period. These components aimed to elucidate the structure–property–function relationship of a β-type Ti-Nb-Zr alloy in dental implant applications. The clinical investigation assessed peri-implant soft tissue conditions, fractal bone characteristics, and adverse events.

Energy-dispersive X-ray spectroscopy confirmed that TNZ implants contained 40.12–41.11% Nb and 7.10–7.49% Zr by weight (Table 1). Nb serves as a β-phase stabilizer, enhancing ductility and strength by refining grain structure, while Zr, a neutral element, contributes to hardening and wear resistance [1]. Mechanical testing demonstrated that TNZ materials exhibited tensile strength, modulus of elasticity, and elongation values comparable to previously reported Ti-13Nb-13Zr alloys [14], and superior to other Ti-Nb-Zr compositions [16]. MG4T materials displayed mechanical properties consistent with those of known cpTi [5]. Fatigue testing indicated that TNZ implants had higher resistance to cyclic loading under the tested conditions than MG4T implants; however, statistical validation was not feasible due to the limited number of specimens. These findings suggest that TNZ alloys may provide enhanced mechanical endurance without a proportional increase in elastic modulus. However, due to the small sample size (*n* = 5), statistical power was limited, and further validation with a larger sample is required.

Despite the observed mechanical differences, corresponding variations in peri-implant bone response were not evident clinically. This discrepancy may result from compensatory biomechanical factors intrinsic to modern implant designs, including platform switching [45,46], tapered geometry [47,48], and surface topography [49,50], which significantly influence load transfer and stress distribution at the bone–implant interface. Surface modification techniques such as ultrasonic rolling have also been shown to enhance both fatigue resistance and antimicrobial performance of TNZ alloys [51]. Under the relatively low and intermittent functional loads typical of dental implant function, these design features may diminish the impact of bulk material stiffness [20,52].

Although several titanium alloys have demonstrated favorable biological and mechanical characteristics [1,12,15], only the Ti-Zr alloy (Roxolid, Straumann, Basel, Switzerland) has achieved widespread commercial adoption [7]. Currently, clinical data regarding Ti-Nb-Zr alloys in dental implantology remain limited. In this study, both TNZ and MG4T implants achieved 100% success rates at 12 months post-loading. These results do not suggest clinical superiority but rather confirm the functional and biological viability of TNZ implants under controlled conditions. The uniformly positive outcomes in both groups are likely attributable to strict enrollment criteria, advanced implant macro-designs, surface treatments, and structured maintenance protocols.

Fractal dimension (FD) analysis was utilized to evaluate changes in trabecular bone adjacent to implants, serving as a complementary approach to conventional marginal bone level (MBL) assessment. Quantitative MBL measurements are constrained by limitations of periapical radiography, such as variations in beam angulation and image distortion, which may lead to discrepancies of 0.5 to 2.5 mm between actual and radiographic measurements [34,35,36,37,38]. As platform-switched implant designs often exhibit minimal or positive MBL changes [46,53], bone changes were qualitatively categorized, and FD analysis was implemented as a more sensitive indicator of trabecular remodeling.

FD analysis has been widely used to characterize trabecular bone patterns [42,54,55] and is recognized for providing reliable data under diverse radiographic conditions [56]. In this study, linear mixed model analysis indicated that changes in FD were significantly influenced by baseline values (Table 9). Group-related differences became apparent only at 12 months post-loading (T3), when a statistically significant but quantitatively small difference was detected. The absence of consistent group differences at earlier time points indicates that FD changes primarily reflect time-dependent remodeling processes rather than immediate material-driven effects. The observed influence of sex at T1 and the lack of age-related associations further suggest that biological variability exerts a modest effect on FD dynamics.

The marginal reduction in FD observed in the TNZ group at 12 months is more appropriately interpreted as a microarchitectural adaptation rather than evidence of pathological bone loss. As all implants maintained marginal bone levels and demonstrated clinical stability according to established implant success criteria [57], the FD changes observed should not be considered pathological. These changes likely reflect a subtle reorganization of trabecular architecture in response to functional loading as reported by Heo et al. [58], aligning with physiologic bone remodeling. Therefore, FD should be regarded as an indirect indicator of bone adaptation rather than a surrogate marker for implant success or failure.

Although customized positioning aids and image-alignment software were employed, interobserver variability in FD measurements persisted, likely due to minor differences in CCD sensor orientation and radiographic angulation. Nevertheless, Bland–Altman analysis indicated acceptable agreement within the 95% limits (Figure 7). The decrease in FD from T0 to T1 may indicate transient biological responses associated with second-stage surgery and flap elevation, both of which are known to affect trabecular density and remodeling dynamics [59,60].

At T3, FD values surrounding TNZ implants were lower than those observed around MG4T implants. This result is initially counterintuitive, as TNZ implants—owing to their lower elastic modulus—are generally expected to reduce stress shielding and promote bone remodeling [1,5,6,12,16]. However, this expectation is rooted in orthopedic paradigms and may not directly translate to dental implantology. In orthopedics, mismatches in elastic modulus are known to cause disuse atrophy and bone resorption under continuous load-bearing conditions [18,19]. By contrast, dental implants operate in a distinct biomechanical context characterized by intermittent, multidirectional, and relatively low-magnitude functional loads [20,21].

Traditionally, elastic modulus mismatch has been considered a key factor in peri-implant stress distribution. However, under oral loading conditions, the bulk material’s stiffness may be secondary to factors such as implant design, surface topography, and biomechanical coupling at the bone–implant interface [20,21]. Unlike orthopedic prostheses that endure sustained axial loads, dental implants are exposed to cyclic, non-continuous loading. In this physiological context, design and interface properties likely play a more dominant role in influencing bone adaptation.

Despite TNZ’s lower elastic modulus, the findings suggest it does not inherently lead to increased trabecular complexity under typical dental loading conditions. Rather than indicating an adverse response, the FD patterns observed may reflect a distinct mode of microarchitectural adaptation shaped by the combined influence of material properties, implant geometry, and loading behavior.

Recent finite element studies have indicated that implants with higher stiffness may distribute loads within a physiologically acceptable range, thereby supporting primary stability and osseointegration [20,22,61]. Furthermore, a meta-analysis reported no significant differences in marginal bone outcomes among Ti, Ti-Zr, and Zr implants, despite substantial differences in elastic modulus [8]. Collectively, these findings challenge the direct clinical relevance of stress-shielding paradigms derived from long-bone orthopedics when applied to dental implantology. The relatively lower FD increase observed around TNZ implants suggests that reduced stiffness does not universally result in enhanced trabecular complexity under oral loading conditions.

No adverse events were attributed to either implant system, indicating a favorable safety profile. Previous mechanical studies have shown that TNZ alloys exhibit cytocompatibility comparable to or greater than that of commercially pure titanium (cpTi) [1,16], and current clinical observations support these findings. Additionally, Ti-Nb-Zr alloys produced by powder metallurgy have demonstrated excellent cytocompatibility and osteoblast viability in vitro [62].

Several limitations of the present study warrant consideration. The modest cohort size and recruitment from only two clinical centers may limit the generalizability of the results. Additionally, FD analysis was based on two-dimensional periapical radiographs, which are prone to projection errors and anatomical overlap. The lack of three-dimensional imaging modalities, such as CBCT, restricts the precise assessment of volumetric bone remodeling. Furthermore, in vitro mechanical fatigue testing was conducted on a limited number of specimens and should be regarded as a comparative screening rather than a definitive evaluation of fatigue limits.

Future research should build upon these findings by conducting larger, multicenter clinical studies with extended follow-up periods and the inclusion of mechanically demanding scenarios. In vivo studies on Ti-Zr-Nb alloys have demonstrated favorable osseointegration and biological safety in animal models, establishing a robust preclinical basis for clinical translation [63]. The incorporation of three-dimensional imaging, advanced texture or fractal analyses, and computational stress modeling may provide further insight into the relationship between implant material properties, mechanical loading, and peri-implant bone adaptation. Such investigations are necessary to determine whether the functional advantages of β-type Ti alloys translate into clinically significant long-term benefits.

## 5. Conclusions

Under the conditions tested, TNZ implants demonstrated superior mechanical endurance and clinical performance comparable to that of MG4T implants over a 12-month functional period. FD analysis revealed no adverse patterns in peri-implant bone microarchitecture, and no implant-related adverse events were reported. These findings support the clinical viability of TNZ as a β-type titanium alloy, combining mechanical resilience with favorable biocompatibility. Nevertheless, longer-term, multicenter clinical trials are warranted to determine whether these advantages persist over time and translate into broader clinical benefit.

## Figures and Tables

**Table 1:** Results of surface composition analysis using energy dispersive X-ray spectroscopy for TNZ implants.

Specimen	Ti (%)	Nb (%)	Zr (%)
1	52.39	40.12	7.49
2	21.99	40.55	7.46
3	51.79	41.11	7.10

% = Weight %.

**Table 2:** Results of tensile testing for the modified Grade 4 cpTi (MG4T) and Ti-Nb-Zr (TNZ) material.

Specimen	Tensile Strength (Mpa)		Modulus of Elasticity (GPa)		Elongation (%)	
	MG4T	TNZ	MG4T	TNZ	MG4T	TNZ
1	1011.2	1131.9	114.8	87.9	23.8	8.6
2	887.2	1031.8	123.5	68.7	23.2	9.9
3	981.6	1132.6	111.3	62.6	23.7	8.5
4	829.2	1307.6	93.1	79.1	26.7	12.2
5	790.8	1091.1	107.3	76.7	27.6	10.8
Mean ± SD	900.0 ± 95.0	1139.0 ± 102.9	110.0 ± 11.2	75.0 ± 9.7	25.0 ± 2.0	10.0 ± 1.6

**Table 3:** Results of fatigue testing for the MG4T and TNZ implants.

Specimen	MG4T Implant	TNZ Implant
1	281,480	1,207,632
2	350,379	1,094,272
3	215,450	1,023,512
4	192,909	876,535
5	232,717	1,110,877
Mean ± SD (cycles)	254,587 ± 56,060	1,062,566 ± 110,015

**Table 4:** Baseline demographic characteristics and implant-related clinical characteristics before loading.

	Test Group (TNZ) (*n* = 38)	Control Group (MG4T) (*n* = 39)	Total (*n* = 77)	*p*-Value
Age				0.3916 ^‡^
N	38	39	77	
Mean (SD)	51.21 (12.18)	53.92 (15.24)	52.58 (13.79)	
Median	53.50	56.00	54.00	
Min, Max	26.00, 71.00	20.00, 93.00	20.00, 93.00	
Sex, N (%)				0.0255 ^$^
Male	20 (52,63)	30 (76.92)	50 (64.94)	
Female	18 (47.37)	9 (23.08)	27 (35.06)	
Arch, N (%)				0.3774 ^$^
Maxilla	11 (28.95)	15 (38.46)	26 (33.77)	
Mandible	27 (71.05)	24 (61.54)	51 (66.23)	
Number of implants placed, N (%)				-
1 implant	38 (100.00)	39 (100.00)	77 (100.00)	
Maximum insertion torque value (Ncm)				0.1721 ^#^
N	38	39	77	
Mean (SD)	41.32 (7.32)	43.67 (6.50)	42.51 (6.97)	
Median	40.00	45.00	45.00	
Min, Max	30.00, 50.00	30.00, 50.00	30.00, 50.00	
Implant stability quotient (ISQ) mean value				
At implant placement (A)				0.0119 ^#^
N	38	39	77	
Mean (SD)	71.39 (10.56)	77.17 (6.59)	74.32 (9.19)	
Median	74.00	78.50	77.00	
Min, Max	43.50, 89.00	56.00, 89.00	43,50, 89.00	
At second-stage surgery (B)				0.3508 ^‡^
N	38	39	77	
Mean (SD)	75.83 (5.52)	77.17 (6.89)	76.51 (6.25)	
Median	76.25	77.00	76.50	
Min, Max	60.00, 85.00	62.50, 94.50	60.00, 94.50	
Change in ISQ values (B-A)				0.0210 ^‡^
N	38	39	77	
Mean (SD)	4.43 (8.93)	0.00 (7.53)	2.19 (8.50)	
Median	3.00	1.00	2.00	
Min, Max	−11.00, 29.50	−16.50, 21.00	−16.50, 29.50	
*p*-value	0.0041 *	1.0000 *	0.0267 *	
Rate of ISQ change: (B-A)/A × 100				0.0535 ^#^
N	38	39	77	
Mean (SD)	8.23 (16.07)	0.50 (10.39)	4.32 (13.96)	
Median	4.03	1.18	2.74	
Min, Max	−13.41, 67.82	−20.89, 37.50	−20.89, 67.82	
*p*-value	0.0026 ^§^	0.8475 ^§^	0.0174 ^§^	

N (%): Number of subjects (percentage). Percentages are calculated based on each group. ^‡^: Independent two-sample *t*-test; ^$^: Chi-square test; -: Not analyzed; ^#^: Wilcoxon rank sum test; *: Paired *t*-test; ^§^: Wilcoxon signed rank test.

**Table 5:** Pocket depth, plaque index, gingival index, and bleeding on probing at 6 months and 12 months after loading.

	Test Group (TNZ) (*n* = 38)	Control Group (MG4T) (*n* = 39)	Total (*n* = 77)	*p*-Value
Pocket depth				
6 months post-loading				0.6654 ^‡^
N	38	39	77	
Mean (SD)	2.39 (0.85)	2.47 (0.75)	2.44 (0.80)	
Median	2.38	2.50	2.50	
Min, Max	1.00, 4.00	1.00, 4.00	1.00, 4.00	
12 months post-loading				0.5808 ^‡^
N	38	39	77	
Mean (SD)	2.62 (0.71)	2.72 (0.86)	2.67 (0.78)	
Median	2.63	2.75	2.75	
Min, Max	1.00, 3.75	1.00, 4.00	1.00, 4.00	
Plaque index				
6 months post-loading				0.4168 ^#^
N	38	39	77	
Mean (SD)	0.24 (0.59)	0.10 (0.31)	0.17 (0.47)	
Median	0.00	0.00	0.00	
Min, Max	0.00, 2.00	0.00, 1.00	0.00, 2.00	
12 months post-loading				0.1869 ^#^
N	38	39	77	
Mean (SD)	0.08 (0.27)	0.21 (0.47)	0.14 (0.39)	
Median	0.00	0.00	0.00	
Min, Max	0.00, 1.00	0.00, 2.00	0.00, 2.00	
Gingival index				
6 months post-loading				0.0779 ^#^
N	38	39	77	
Mean (SD)	0.13 (0.47)	0.00 (0.00)	0.06 (0.34)	
Median	0.00	0.00	0.00	
Min, Max	0.00, 2.00	0.00, 0.00	0.00, 2.00	
12 months post-loading				0.0454 ^#^
N	38	39	77	
Mean (SD)	0.00 (0.00)	0.10 (0.31)	0.05 (0.22)	
Median	0.00	0.00	0.00	
Min, Max	0.00, 0.00	0.00, 1.00	0.00, 1.00	
Bleeding on probing				
6 months post-loading, N (%)				-
Positive	0 (0.00)	0 (0.00)	0 (0.00)	
Negative	38 (100.00)	39 (100.00)	77 (100.00)	
12 months post-loading, N (%)				-
Positive	0 (0.00)	0 (0.00)	0 (0.00)	
Negative	38 (100.00)	39 (100.00)	77 (100.00)	

N (%): Number of subjects (percentage). Percentages are calculated based on each group. ^‡^: Independent two-sample *t*-test; ^#^: Wilcoxon rank sum test; -: Not analyzed.

**Table 6:** Descriptive statistics of fractal dimensions.

		Overall	Observer 1		Observer 2		
			Control (MG4T)	Test (TNZ)	Control (MG4T)	Test (TNZ)	
Time	N	154	39	38	39	38	*p*-Value
T0	1.34 (0.09)		1.35 (0.10)	1.34 (0.10)	1.34 (0.07)	1.33 (0.09)	0.899
T1	1.30 (0.09)		1.33 (0.09)	1.34 (0.08)	1.28 (0.08)	1.26 (0.09)	<0.001
T2	1.33 (0.09)		1.38 (0.07)	1.35 (0.10)	1.30 (0.08)	1.28 (0.09)	<0.001
T3	1.33 (0.10)		1.38 (0.09)	1.34 (0.09)	1.32 (0.09)	1.27 (0.09)	<0.001

Note. Mean (Standard deviation); T0: Second-stage surgery; T1: Loading; T2: 6 months post-loading; T3: 12 months post-loading.

**Table 7:** Agreement between two observers.

Time	ICC	
T0	0.558	0.383 < ICC < 0.694
T1	0.16	−0.037 < ICC < 0.353
T2	0.177	−0.031 < ICC < 0.377
T3	0.176	−0.029 < ICC < 0.375

ICC: Intraclass correlation coefficient; T0: Second-stage surgery; T1: Loading; T2: 6 months post-loading; T3: 12 months post-loading.

**Table 8:** Paired comparisons of fractal dimensions between each time point and the baseline.

Observer	Time	Group	Mean (SD)	*p*-Value *
1	T1–T0	1	−0.02 (0.09)	0.119
1	T1–T0	2	−0.00 (0.10)	0.829
1	T2–T0	1	0.03 (0.12)	0.120
1	T2–T0	2	0.01 (0.11)	0.597
1	T3–T0	1	0.02 (0.11)	0.335
1	T3–T0	2	0.00 (0.12)	0.984
2	T1–T0	1	−0.06 (0.08)	<0.001
2	T1–T0	2	−0.07 (0.13)	0.001
2	T2–T0	1	−0.04 (0.10)	0.017
2	T2–T0	2	−0.05 (0.12)	0.013
2	T3–T0	1	−0.03 (0.10)	0.076
2	T3–T0	2	−0.07 (0.15)	<0.001

*: Paired *t*-test; Group 1: Control group (MG4T); Group 2: Test group (TNZ); T0: Second-stage surgery; T1: Loading; T2: 6months post-loading; T3: 12 months post-loading.

**Table 9:** Linear mixed model analysis of fractal dimension changes over time and covariates relative to baseline (T0).

Group	Characteristic	Beta	95% CI	*p*-Value
T1 vs. T0	baseline value	−0.665	−0.8168, −0.5131	<0.001
	factor (group)			
	1	—	—	
	2	0.0011	−0.0260, 0.0282	>0.9
T1 vs. T0 covariate	baseline value	−0.6804	−0.8320, −0.5288	<0.001
	factor (group)			
	1	—	—	
	2	−0.0076	−0.0353, 0.0201	0.6
	age	−0.0004	−0.0014, 0.0006	0.4
	factor (sex)			
	1	—	—	
	2	0.0345	0.0051, 0.0638	0.022
T2 vs. T0	baseline value	−0.8289	−0.9921, −0.6656	<0.001
	factor (group)			
	1	—	—	
	2	−0.0234	−0.0528, 0.0061	0.12
T2 vs. T0 covariate	baseline value	−0.832	−0.9976, −0.6665	<0.001
	factor (group)			
	1	—	—	
	2	−0.0278	−0.0584, 0.0029	0.075
	age	−0.0004	−0.0015, 0.0007	0.4
	factor (sex)			
	1	—	—	
	2	0.015	−0.0175, 0.0475	0.4
T3 vs. T0	baseline value	−0.8697	−1.0484, −0.6911	<0.001
	factor (group)			
	1	—	—	
	2	−0.0404	−0.0730, −0.0078	0.016
T3 vs. T0 covariate	baseline value	−0.8726	−1.0538, −0.6913	<0.001
	factor (group)			
	1	—	—	
	2	−0.0432	−0.0774, −0.0090	0.014
	age	−0.0001	−0.0014, 0.0011	0.8
	factor (sex)			
	1	—	—	
	2	0.0106	−0.0248, 0.0461	0.6

CI = Confidence Interval; T0: Second-stage surgery; T1: Loading; T2: 6 months post-loading; T3: 12 months post-loading; Group 1: Control group (MG4T); Group 2: Test group (TNZ); Sex 1: male; Sex 2: female. For categorical variables, level 1 is the reference category, and “—“ indicates that no coefficient is estimated for the reference.

## Data Availability

The original contributions presented in the study are included in the article, further inquiries can be directed to the corresponding authors.

## Abbreviations

AE	adverse event
ASTM	American Society for Testing and Materials
BIC	bone-implant contact
BOP	bleeding on probing
CBCT	cone-beam computed tomography
cpTi	commercially pure titanium
FD	fractal dimension
GI	gingival index
ISQ	implant stability quotient
MBL	marginal bone level
MG4T	modified Grade 4 commercially pure titanium
Nb	niobium
PA	periapical radiograph
PD	probing depth
PI	plaque index
SEM	scanning electron microscopy
TNZ	titanium-niobium-zirconium
Zr	zirconium
